# Institutional Needs

**Published:** 1916-03-11

**Authors:** 


### Institutional Needs.

### THE CAMPLING LEG SPLINT.

(Mayer and Meltzer, 71 Great Portland Street, W.)

This new back splint with footpiece embodies a simple but ingenious idea which should have been thought of before, but apparently never has been. The footpiece, instead of being rigidly fixed at a right angle to the splint, is hinged and provided with a rack whereby it can be fixed at various angles from 90° to 180° with the main leg splint itself. There are distinct and marked advantages derived from this arrangement. To take a minor one first, the whole splint will pack flat, and so space is economised. More important than this, the sole of the patient's foot and the back of his heel (which fits into a window in the splint in the usual way) can be inspected, washed, dressed, or treated *without disturbing the leg* and without even unfastening the bandages which fix it to the splint. This device, as any experienced surgeon or nurse will appreciate, facilitates the treatment of sore heels when they have occurred, and their prevention as well—an even more excellent property. The splint is sold in three sizes, price 7s. 6d. each, or 21s. the set of three.

